# Autism Through the Ages: A Mixed Methods Approach to Understanding How Age and Age of Diagnosis Affect Quality of Life

**DOI:** 10.1007/s10803-021-05235-x

**Published:** 2021-09-04

**Authors:** Gray Atherton, Emma Edisbury, Andrea Piovesan, Liam Cross

**Affiliations:** 1 Department of Psychology, Edge Hill University, St Helens Rd, Liverpool, Ormskirk, L39 4QP Lancashire UK; 2 Università Iuav di Venezia, 30135 Venice, VE Italy

**Keywords:** Autism, Adulthood, Mental health, Autism quotient, Quality of life, Mixed methods

## Abstract

A significant proportion of autistic adults today were not diagnosed until later in life, a group referred to as the ‘lost generation,’ which may affect mental health. In Study 1 we explored quality of life and autistic trait levels in 420 autistic and TD adults, and in Study 2 we explored the experiences of 8 autistic adults diagnosed as adults. We found that autistic adults had lower quality of life outcomes and higher autistic trait levels which related to age of diagnosis, and qualitative findings indicated that while adults were empowered by their new diagnosis, they still require specialized supports. Our findings are discussed, emphasizing future directions and implications for the current care system in place for autistic adults.

**Supplementary Information:**

The online version contains supplementary material available at 10.1007/s10803-021-05235-x.

Research suggests that as we get older, we gain social confidence (Miner-Rubino et al., 2004). We experience fewer highs and fewer lows, our ideal self is closer to our actual self, and we have less volatility in our social networks (Diener et al., 1999; McAdams et al., 1993; Segrin, 2003). Theorists such as Erikson (1994) suggest that as we age, we gain more positive feelings about the self after we form a coherent identity during adulthood (Arnett, 2007).

Understanding this trajectory in the autistic population is essential. Some research suggests as autistic adults age, symptoms improve or remain stable (Happé & Charlton, 2012; Howlin et al., 2014; Magiati et al., 2014; Siebes et al., 2018). However, research is unclear as to why symptoms may improve. Some suggest that targeted therapies early in life may improve symptoms (Fein et al., 2013). However, what constitutes ‘improvement of symptoms’ is a complex issue that depends on what constitutes improvement to given individuals. For instance, many suggest that over time autistic people develop the ability to ‘mask’ or ‘camouflage’ symptoms to appear ‘normal’ (Hull et al., 2017). Compensation in this way, however, is not necessarily positive or indicative of ‘improvement.’ While it may benefit an autistic person in some spheres, for most, the costs of camouflaging outweighs the rewards (Mandy, 2019). For those who mask regularly, there is an increased risk for developing co-occurring conditions such as anxiety and depression (Cage et al., 2018). This may explain why autistic adults are at an increased risk of experience psychological symptoms and distress compared to the general population as they age (Lever & Geurts, 2016), which can negatively influence the trajectory of quality of life (QoL).

QoL is defined by the World Health Organization (WHO) as ‘an individual’s perception of their position in life in the context of the culture and value systems in which they live (World Health Organization & Division of Mental Health and Prevention of Substance Abuse, 1997, p. 1). QoL encompasses and is affected by many factors, including loneliness, social anxiety/avoidance, social support, and satisfaction with life. Research suggests the above constructs significantly affect the quality of life in autistic and non-autistic adults. Specifically, social anxiety and loneliness have been found to mediate the relationship between autistic traits and quality of life (Reed et al., 2016), as has perceived social support (Stimpson et al., 2020). Satisfaction with life is a more unidimensional and highly subjective measure of wellbeing, which previous research has found to be lower in autistic adults in relation to social support (Schmidt et al., 2015). Research shows that perceived stress (Hong et al., 2016) and reduced social support (Kapp, 2018) negatively influence QoL for autistic adults.

Autistic females may be particularly at-risk for developing poor QoL outcomes (Mason et al., 2018). Autistic females reportedly show higher social motivation and engage in more traditional friendships than autistic males (Sedgewick et al., 2016), but this is not necessarily protective. Compared to males, they are at an increased risk of developing internalizing disorders, which negatively impact upon QoL (Jamison & Schuttler, 2015; Solomon et al., 2012). They are also at a higher risk for suicidality following bullying (Holden et al., 2020), indicating a possible increased sensitivity in autistic females relating to social rejection. For instance, Greenlee et al. (2020) found that while both male and female autistic adolescents experienced peer victimization, female experiences of victimization more heavily factored into internalizing symptoms such as anxiety and depression. The authors suggest that this may be due to the more nuanced, language-dependent social contexts in which female friendships form, which may disproportionately disadvantage autistic females when engaging with peers.

The timing of an autistic diagnosis is critical in that access to diagnostic services and formal support systems enhance the QoL of autistic adults (Renty & Roeyers, 2006). Renty and Roeyers’s (2006) study on linking QoL with formal services was interesting in that it was the perception of services and supports, rather than the actual usage of these supports, that predicted wellbeing in autistic adults. Receiving a diagnosis may also help reduce the urge to mask autistic symptoms (Bradley et al., 2021). Once diagnosed, autistic people feel more comfortable not conforming to neurotypical behavioral expectations. In this sense, it may be that there are aspects of identifying as autistic, and being made aware of the supports available, that are in and of themselves tied to QoL. This suggests that a diagnosis in and of itself, irrespective of actual usage of services, may help improve wellbeing.

Many adults are only now receiving autism diagnoses, termed the ‘lost generation’ of autistic adults (Lai & Baron-Cohen, 2015). Some of this may be due to camouflaging or compensation as well as changes to diagnostic procedures in recent years. Research suggests that severity of impairments influences the timing of an autism diagnosis; those diagnosed later often have subtler symptoms that lead to missed identification (Mandell et al., 2005). Borrowing from Erickson’s theory of lifespan development, receiving a diagnosis such as autism plays a critical role in subsequent identity formation, allowing individuals to refine their experiences into a unified sense of self and use personal knowledge to explore possible futures (Arnett, 2007).

Though autistic adults who have received a diagnosis later in life primarily report it as a positive experience (Leedham et al., 2020; Lewis, 2016), the journey to diagnosis later rather than earlier in life may have resulted in early identity formational experiences that were sub-optimal. Furthermore, understanding how navigating a new identity, including understanding the self and being understood by others in light of a newly received diagnosis, may be particularly stressful. For these reasons, autistic adults only now receiving a diagnosis may have reduced quality of life. Specifically, it may be that living without a diagnosis or with a misdiagnosis can contribute to anxiety and depression stemming from a lack of self-understanding that comes with an accurate diagnosis (Zener, 2019) and a missed opportunity for many years to perceive the availability of social supports.

To explore this, we conducted two studies. Study 1 details a quantitative investigation of 420 adults, half of whom were diagnosed as autistic and half of whom were typically developed (TD). In line with Erickson’s theory of life span development, it was hypothesized that QoL measures and age would be positively related in the TD population. In contrast, it was predicted that this trajectory would not follow in autistic adults, many of whom comprise the ‘lost generation’ of adults having received late diagnoses. We hypothesized that this relation would not be found because many autistic adults are only now being diagnosed and thus did not experience supports or acceptance earlier in life that is crucial to those with ASC (Autism Spectrum Condtion). To account for this, we specifically predict that among autistic individuals, age of diagnosis plays a more critical role than chronological age in QoL outcomes and in symptom trajectory, with a higher age of diagnosis leading to poorer social QoL outcomes and higher levels of self-reported autistic traits. Study 2 then follows up on these findings using qualitative methods to explore identity, QoL and the impact of a late diagnosis in eight autistic adults. Using interpretive phenomenological analysis (IPA) (Smith & Shinebourne, 2012), four themes were uncovered, all of which present the complexities of reconceptualizing the self as autistic and having spent a lifetime without that knowledge.

## Study 1 Methods

### Participants

Four hundred and twenty people took part in this study recruited via Prolific (Oxford, UK). We recruited 210 TD individuals (118 females and 92 males; mean age = 34.13, SD = 13.45) and 210 individuals with Autism Spectrum Condition (ASC) (108 females and 102 males; mean age = 29.03, SD = 10.53). Participants were paid in line with UK minimum wage for time spent filling out the survey, which took on average 30 min. The ethics committee granted ethical approval for this study at Edge Hill University. Each participant gave consent to participate in this research.

### Design, Materials & Procedure

First, participants reported whether they had a diagnosis of autism. If yes, they were then asked whether this diagnosis was given to them by a medical professional and at what age they received the diagnosis. The ability to provide this information is highly correlated with the actual existence of diagnosis (Daniels et al., 2012). It has been used in similar online studies of autistic adults to confirm diagnoses (Baron-Cohen et al., 2015). Next, participant demographic information was recorded, followed by several self-report questions measuring aspects of QoL.

Specifically, participants completed the UCLA Loneliness scale (UCLA-LS, Russell et al., 1980), a 20 item measure scored on a 4-point Likert scale measuring subjective feelings of loneliness and social isolation (i.e. ‘I cannot tolerate being so alone’, ‘I feel left out’). followed by the Liebowitz Social Anxiety Scale (LSAS, Liebowitz, 1987), a 24 item measure in which participants rank their respective anxiety and avoidance of certain social behaviors (i.e. ‘Telephoning in public’; ‘Meeting strangers’) on a 4-point Likert scale. The next measure was the Multidimensional Scale of Perceived Social Support (MSPSS, Zimet et al., 1988), a 12 item scale measured on a 7-point Likert that rates a person’s relationships with family, friends and significant others (i.e. ‘my family really tries to help me’; ‘There is a special person who is around when I am in need’). Followed by the Satisfaction with Life Scale (SLS, Diener et al., 1985), a 5 item measure rated on a 7-point Likert, which asks people to rate their overall contentedness with their life (i.e. ‘In most ways my life is close to my ideal’).

Finally, participants completed the Autism Quotient (AQ) (Baron-Cohen et al., 2001) to measure autistic traits. In the AQ, participants reported their agreement with 50 statements indicating autistic traits (i.e., ‘I prefer going to the library than a party’) using a 4-point Likert scale. The entire Likert scale was used to form AQ scores rather than the Baron-Cohen et al. (2001) original dichotomous response method. Using this method of scoring increases possible max scores from 50 to 200. Likert scoring the AQ has been shown to more adequately retain the detail in responses, increase the variability in scores and increase the overall reliability and validity of the measure (Stevenson & Hart, 2017). Research on the AQ using the Likert scoring system suggests that the middle 50% of the reported mean scores range from 102.42 to 108.10 (Stevenson & Hart, 2017). The dichotomous scoring method was also used to identify and exclude anyone in the TD sample who possesses a clinical level of autistic traits (this is defined as anyone scoring above 31, Baron-Cohen et al., 2001). This was done to account for the possibility that someone with autism who was undiagnosed ended up in our TD group. Essentially the dichotomous score allowed us to identify anyone without a known autism diagnosis but who displayed traits at or above the clinical level in order to exclude them from the TD group. For all other results the total of the full continuous Likert scale was used which gives a score of 50–200.

### Data Analysis

Four subjects were not included from the original 420 participants because they did not reach the end of the online questionnaire. Additionally, 17 individuals from the TD group who had dichotomous AQ scores in the clinical range were also excluded. The analyses reported here refer to the remaining 399 participants (191 = TD, 208 = ASC).

Following the standard scoring procedures, we calculated a (i) Loneliness score from the UCLA-LS, a (ii) Social Anxiety score and a (iii) Social Avoidance score from LSAS, a (iv) Social Support score from MSPSS, and a (v) Life Satisfaction score from SLS. Reliability analysis was conducted between these five scores to test the possibility of combining them into a single Quality of Life measure (QoL). First, the Loneliness, Social Anxiety and Social Avoidance scores were reversed by subtracting each participant’s score from the maximum possible score. In this way, a higher score was associated with a higher quality of life on all measures; a higher score was associated with lower loneliness, social anxiety and avoidance, and higher social support and life satisfaction. The five measures were all highly intercorrelated (all *r*_s_s > .30 and *p*s < .0001), and Cronbach’s alpha showed the five measures to reach acceptable reliability, α = 0.81.

This QoL score was calculated for each participant as follows; z scores were calculated for Social Support, Life Satisfaction and the reversed Loneliness, Social Anxiety and Social Avoidance. The five z scores were then averaged, and the result was the combined QoL score used in the following analyses. Participants scored between − 1.95 and 1.59, with a higher score indicating a higher quality of life. QoL highly correlated with the original five measures (all *r*_s_s > .72 and *p*s < .0001), and a Cronbach’s alpha including the five measures and QoL reached acceptable reliability, α = 0.83. Analysis of the QoL composite score was consistent with analyzing the five scores separately, which can be found in the Supplementary Materials, along with further details for all measure checks.

## Study 1 Results

Table 1 shows descriptive’s for age, age of diagnosis, AQ and QoL divided between ASC and TD and by sex. All variables except QoL were abnormally distributed as indicated by the Kolmogorov Smirnov tests reported in Table 1. Non-parametric statistical tests were therefore used where available.

**Table 1:** Median and range of age, age of diagnosis, AQ and QoL

Variable	Kolmogorov Smirnov test	ASC		TD	
		Male	Female	Male	Female
*N*		101	107	78	113
Age	KS (399) = .13, *p* < .001	24 (18–57)	27 (15–63)	26.5 (16–73)	33 (18–79)
Age of diagnosis	KS (208) = .13, *p* < .001	15 (2–54)	21 (3–62)		
*AQ*	KS (399) = .09, *p* < .001	139 (91–188)	153 (99–194)	119 (91–141)	108 (77–137)
*QoL*	KS (399) = .04, *p* = .16	− 0.29 (− 1.85 to 1.44)	− 0.55 (− 1.95 to 1.48)	0.48 (− 1.06 to 1.59)	0.42 (− 1.10 to 1.59)

Descriptive statistics suggested that females (191 = TD, 208 = ASC) were diagnosed with autism later than males, which was confirmed with a Mann Whitney U test (U(N_male_ = 101, N_female_ = 107) = 7390.00, z = 4.58, *p* < .001). Inspection of Table 1 also suggested that women were more likely to be diagnosed with autism in adulthood (18+ years), while men were more likely to be diagnosed with autism in childhood (< 18 years). We tested this possibility by dividing both autistic males and females into groups based on whether they were diagnosed before or after 18 (Table 2). A Pearson’s Chi-Square was then conducted to assess the association between sex and age of diagnosis. There was a significant association between sex and age of diagnosis (*χ*^*2*^(1) = 12.24, *p* < .001); men were indeed more likely to be diagnosed with autism during childhood, while women were more likely to be diagnosed during adulthood.

**Table 2:** Number of participants diagnosed before 18 years or later as function of sex

Age of diagnosis	< 18	18+
Male	66 (65.3%)	35 (34.7%)
Female	44 (41.1%)	63 (58.9%)

**Fig. 1:**
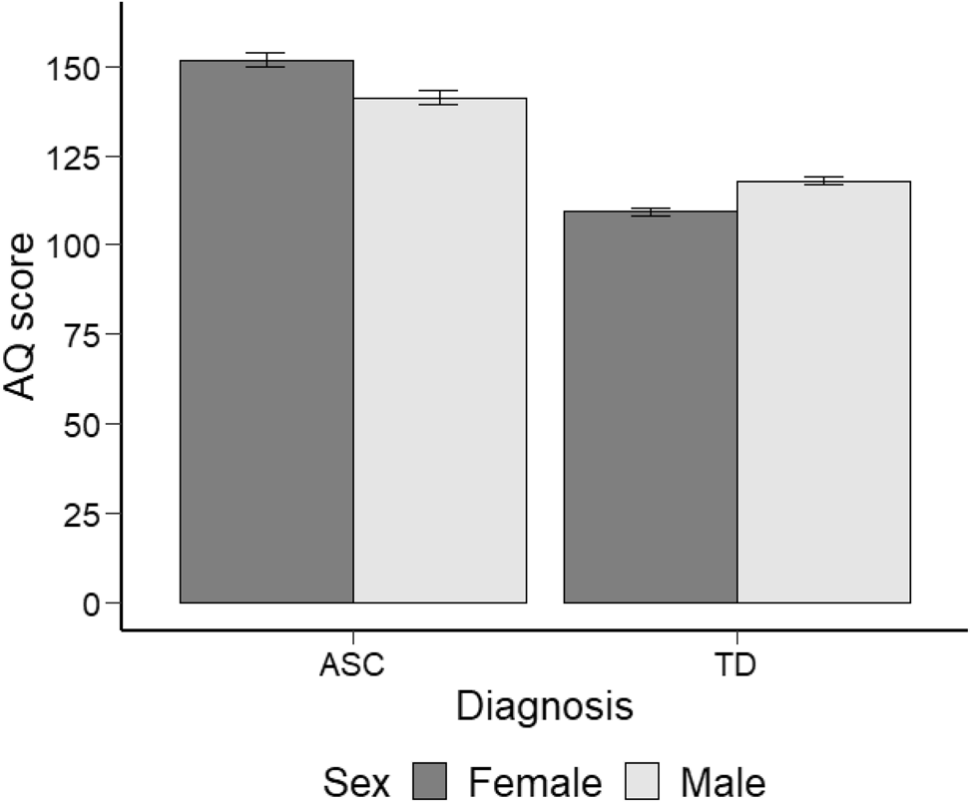
AQ scores of participants divided between ASC and TD and by sex

We explored the effect of diagnosis and sex on AQ and QoL. A two-way ANOVA was conducted with diagnosis (ASC and TD) and sex (male and female) as a between-subjects factor (the F-test is robust to violations of normality in terms of Type I error Blanca et al., 2017; Schminder et al., 2010). As it is possible to see from the coefficients of the ANOVAs reported on Table 3, there was no significant main effect of sex on AQ, but there was a significant main effect of diagnosis on AQ. Post-hoc tests showed that AQ scores were significantly higher in the ASC group than in the TD group. There was also a significant interaction effect between diagnosis and sex on inspection of Fig. 1. Mann Whitney U tests reported in Table 3 indicated that females had higher AQ scores in the ASC group and lower AQ scores in the TD group compared to males. There was a significant main effect of diagnosis on QoL, and post-hoc tests showed that QoL scores were significantly lower in the ASC group than in the TD. However, there was no significant main effect of sex on QoL, and there was no significant interaction between diagnosis and sex on QoL.

**Table 3:** Inferential statistics (ANOVAs with post-hocs and spearmans correlations)

Participants	Statistics	Variable	AQ	QoL
All	ANOVA main effect	Diagnosis	***F* = 386.22, *η***_***p***_^***2***^ = .49*	***F* = 144.55, *η***_***p***_^***2***^ = .27*
All	Post-hoc	Diagnosis	[26.63, 36.22]*	[3.35, 4.65]*
All	ANOVA main effect	Sex	*F* = .28, *η*_*p*_^*2*^ = .001	*F* = 1.43, *η*_*p*_^*2*^ < .01
All	Post-hoc	Sex		
All	ANOVA interaction effect	Diagnosis * sex	***F* = 3.13, *η***_***p***_^***2***^ = .08*	*F* = 1.02, *η*_*p*_^*2*^ < .01
TD	Mann Whitney U test	Sex	**U(Nm = 78, Nf = 113) = 2698.00, z = **− 4.55*	
ASC	Mann Whitney U test	Sex	U(Nm = 101, Nf = 107) = 7084.50, z = 3.88*	
TD	Spearman’s correlation	Age	***r***_**s**_** = **− .29*	***r***_**s**_ = .35*
ASC	Spearman’s correlation	Age	***r***_**s**_ = .23*	*r*_s_ = − .03
ASC	Spearman’s correlation	Age of diagnosis	***r***_**s**_ = .44*	***r***_**s**_** = **− **.21**^**┼**^

Bold is used to further illustrate significance

NB: all *F*s had (1, 395) degrees of freedom; All post-hocs were Bonferroni corrected

*p < .001; ^**┼**^*p* = .002

In summary, ASC individuals had higher autistic traits and lower quality of life compared to TD individuals. Furthermore, autistic women had higher levels of autistic traits compared to autistic men, while the opposite trend was found among TD males and females. Finally, men and women did not have significantly different QoL scores.

**Fig. 2:**
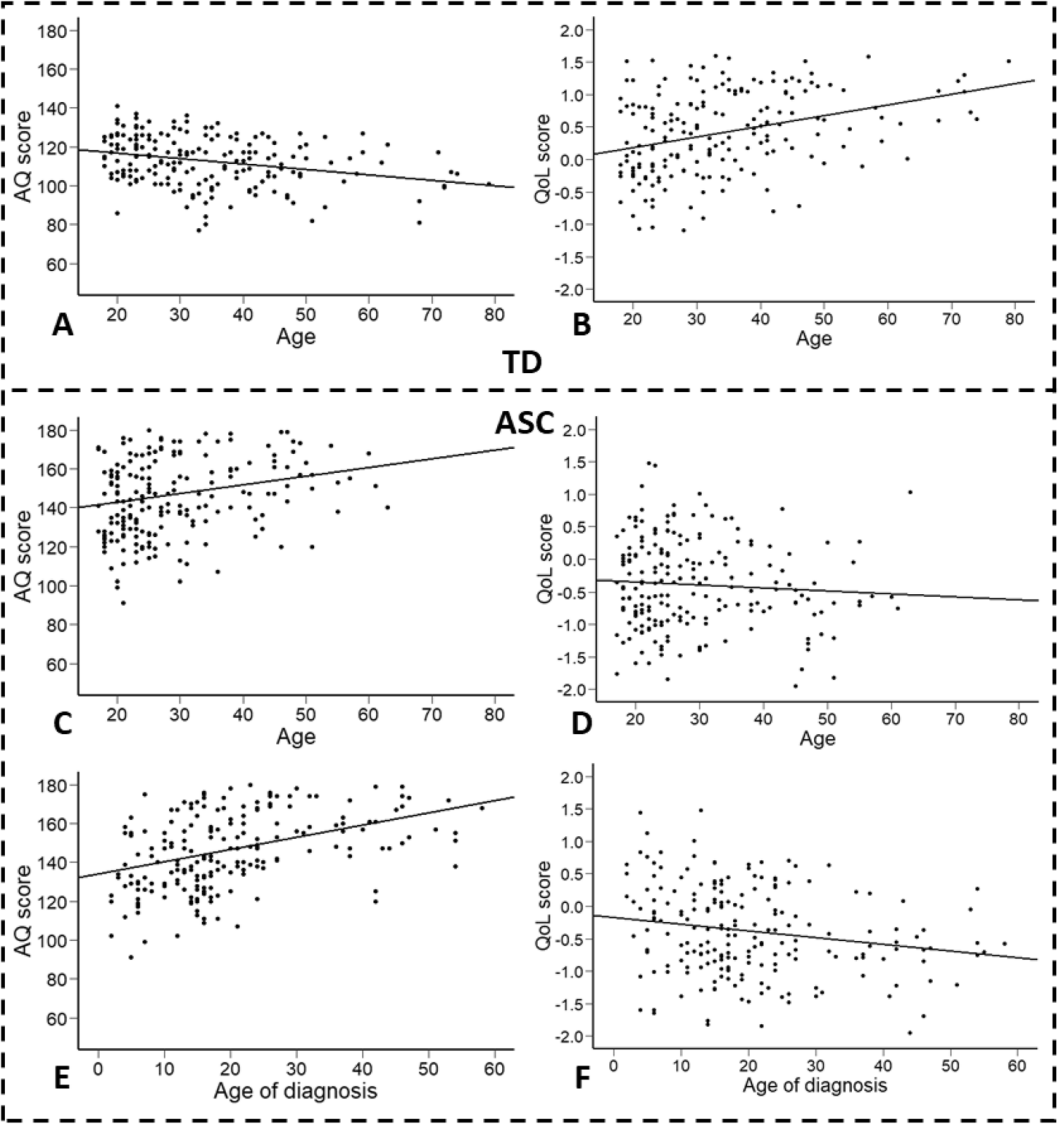
Scatter plots showing the relationship between age, AQ and QoL Scores in the TD Group (Top Panels) and in the ASC Group (Middle Panels), and the relationship between age of diagnosis, AQ and QoL scores in the ASC group (Bottom Panels). Panel **A** Relationship between age and AQ scores in the TD group. Panel **B** Relationship between age and QoL scores in the TD group. Panel **C** Relationship between age and AQ scores in the ASC group. Panel **D** Relationship between age and QoL scores in the ASC group. Panel **E** Relationship between age of diagnosis and AQ scores in the ASC group. Panel **F** Relationship between age of diagnosis and QoL scores in the ASC group

Two-tailed Spearman’s correlations were conducted to explore whether participants’ age was related to AQ and QoL. Correlations were performed separately for those with a diagnosis of ASC and TDs to test whether older participants in both the ASC and TD groups reported a higher quality of life (see Table 3 for Spearman’s coefficients and Fig. 2, panels A-D). In the TD group, there was a significant negative correlation between age and AQ and a significant positive correlation between age and QoL. In contrast, there was a significant positive correlation between age and AQ in the ASC group and no significant relationship between age and QoL. This suggested that while ageing was related to lower autistic traits and higher quality of life in typically developed people, ageing was related to higher autistic traits but not the quality of life for autistic people. Within the ASC group, two-tailed Spearman’s correlations were also performed to explore whether the age of diagnosis was related to AQ and QoL (see Table 3 and Fig. 2, panels E and F). There was a significant positive correlation between diagnosis age and AQ and a significant negative correlation between diagnosis age and QoL. The findings suggested that, although the age of autistic people was not related to their quality of life, their diagnostic age was, with a later diagnosis associated with a poorer quality of life.

## Study 1 Discussion

Study 1 suggested that autistic adults experience lower QoL than TDs, as has been found by other studies (McDonald, 2020; van Heijst & Geurts, 2015). However, in contrast to other research (van Heijst & Geurts, 2015), autistic adults in our study were found to have a different developmental trajectory than TDs regarding age and its relation to QoL outcomes. While van Heijst and Geurts (2015) found that older adults with and without ASC had similar quality of life patterns over the lifespan, our study suggests that autistic and TD adults follow different QoL trajectories.

While TD samples showed higher QoL scores and lower autistic traits commensurate with higher age, this pattern did not hold for autistic participants. Our autistic sample had higher AQ scores commensurate with age, calling into question whether autistic symptomology does indeed ‘improve’ over the lifespan. We hypothesized that the age of diagnosis played a critical role, and delays in receiving a diagnosis would have adverse effects on the social development of autistic adults across the lifespan. In line with our hypothesis, age of diagnosis was an essential factor for explaining QoL. Specifically, age of diagnosis correlated with increased social anxiety, social avoidance, and a lack of social support. Furthermore, the older their age, the higher their AQ score in our autistic sample, while the reverse was true for TDs. This finding calls into question whether those diagnosed later in life have subtler autistic symptoms, as elevated AQ scores in this subset of the sample suggest the opposite.

As predicted, the age of diagnosis was significantly related to QoL; adults diagnosed earlier in life had better QoL outcomes, while those diagnosed later had poorer outcomes. Thus, it appears that early identification and diagnosis of autism may be critical to the social success and mental health of autistic adults. This has been suggested by previous qualitative research in which adults following diagnosis express relief, improved social strategies, self-acceptance, and a formation of an autistic identity helped by connection to autistic peers (for a review, see (Huang et al., 2020).

Gender also played a critical role with regards to participant experiences. Similar to other studies, we found that males received a diagnosis of autism several years earlier than females (Krahn & Fenton, 2012), putting females at a greater risk of remaining undiagnosed and ineligible for clinical support (Dworzynski et al., 2012). While both males and females mask symptoms, females may engage in more compensatory masking (Wood-Downie et al., 2021), though other studies do not find an effect of gender (Livingston et al., 2020). Future work will want to investigate how gender affects missed diagnoses for women. Hull et al. (2020) note that there may be a specific female autistic phenotype that current diagnostic procedures are not tuned to recognizing, as they are based predominantly on male populations and symptom presentation. Autistic females may, for instance, have restricted interests that are not initially recognized as ‘autistic’ in that they are more conventional (such as interest in fictional characters or animals) (Grove et al., 2018).

Wider systematic gender issues may also explain the lack of understanding of a female autistic phenotype in relation to how men and women are expected to communicate and interact within society. Females are commonly viewed as being more empathetic and communal (Hess et al., 2000; Kite et al. 2008), all of which are traits that are at odds with commonly held autism stereotypes, such as individuals who are withdrawn and socially difficult (Wood & Freeth, 2016). Being misunderstood in relation to autistic stereotypes and perhaps even gender stereotypes may itself explain why autistic females rated themselves as having higher autistic trait expressions relative to autistic males; it may be that pressures to conform to female standards of social conduct increase self-perceived autism severity in female versus male autistic people.

It is clear from the pattern of results that autistic adults are continuing to face challenges across the lifespan compared to TDs, and that age of diagnosis plays a role in this disparity. Poorer outcomes for adults who were diagnosed later in life are in many ways a rebuke of the idea that those who are not identified early in life may be individuals with less severe symptoms. In our sample, people who were diagnosed later had higher self-reported traits and poorer outcomes. This instead suggests that autistic people late to receive a diagnosis may not necessarily be presenting differently, but that there may be structural inequalities that limit their ability to obtain an early diagnosis, such as culture, gender, ethnicity or socioeconomic status, all of which can contribute to a delayed diagnosis (Mazurek et al., 2019). Continuing to understand how an autism diagnosis can be missed, including implicit biases that lead to underdiagnosed groups, and how to improve access to diagnostic and therapeutic services, perhaps through the initial use of self-report measures like the AQ, would be an important area of continued work. In light of this, Study 2 is a qualitative study in which we interviewed eight autistic adults, all of whom were diagnosed later in life, to understand better the unique challenges those with a late diagnosis face.

## Study 2 Methods

To further understand the lived experiences of autistic adults with a late diagnosis, we undertook a qualitative investigation. Using a semi-structured interview format, one researcher interviewed eight autistic adults (4 males, 4 females) about their late-stage diagnosis experiences. These eight participants were recruited from Study 1. Specifically, participants who received an diagnosis over the age of 18 were emailed asking if they would like to participate in an interview about their experiences receiving a diagnosis of autism in adulthood. All of those who agreed to be interviewed within the 2-week time frame of data collection were interviewed. Please see Table 4 for participant demographics. All interviews were conducted over the phone/online communication platforms and lasted on average 1 h. Our interview protocol can be found in supplementary materials. All interviewees were compensated £10. The ethics committee granted ethical approval for this study at Edge Hill University. Each participant gave consent to participate in this research and for their quotes to be used. Additionally each participant was invited to review and comment on qualitative analysis and exclude any quotes as part of a member check pre-publication.

**Table 4:** Participant demographics, including gender, age, age of diagnosis, ethnicity and country of birth

Gender	Age	Age of diagnosis	Ethnicity	Country of birth
Female	49	39	White	UK
Female	38	25	White	Czech Republic
Female	24	24	White	UK
Female	63	61	White	UK
Male	34	30	White	Germany
Male	54	52	White	UK
Male	31	25	White	Sweden
Male	26	26	White	UK

## Study 2 Results

A semi-structured interview protocol was developed following the formula of Atherton et al. (2018). Please see the supplementary materials for the complete protocol. Interpretive phenomenological analysis (Smith & Shinebourne, 2012) was used to analyze the qualitative data. In qualitative research using IPA, participants are encouraged to take the discussion into new places so that the interview reflects a participant’s unique values, beliefs and experiences. This is a central aspect of IPA, which has been found to be particularly pertinent to autism research. IPA's fluidity and person-centred approach align well with participatory research designs, a growing movement within autism studies, emphasizing lived experience and addressing the needs of the autistic community (MacLeod, 2019).

Following the interviews' transcriptions, two separate researchers independently coded the interviews. They later convened to agree on the sub-themes for each interview consensually, following the process used by Atherton et al. (2018). Two researchers independently coded for salient patterns found within the text, which they then independently grouped into subthemes for each interview, in line with Graneheim and Lundman (2004). They then convened and for each interview agreed upon the subthemes present in each interview. Through this process, they created a master list of subthemes that ran throughout all the interviews. Then, the researchers decided upon master themes that encapsulated the subthemes. Once this was complete the member check was undertaken, and a list of all subthemes and master themes were sent to all participants. Five out of the eight interviewees responded with comments. Overall, member checks revealed an agreement with our understanding and interpretation of the interviews, though there were some areas they felt could be clarified. These have been reflected in the final themes. A full list of the subthemes, master themes and the frequency counts can be found in Table 5. The four themes were barriers to a diagnosis, negatives of a non-diagnosis, benefits of a diagnosis and two sides to a diagnosis. While some indicative quotes are presented in text a more extensive list of relevant quotes and examples per theme and sub theme can be found on the themes table in the supplementary mateirals.

**Table 5:** Frequency count of subthemes within interview data

Themes/subthemes	Frequency of interviews	Frequency of theme/subtheme
*Barriers to a diagnosis*	8	61
Academic and social success	7	19
Perceptions and Stigma	6	24
Gender (for females)	4	6
Long, difficult process	5	12
Negatives of a non-diagnosis	8	60
Feeling like an alien	3	6
Not fitting in	7	13
Bullying/exclusion	4	7
Masking/camouflaging	8	22
Mental health strains	6	12
*Benefits of a diagnosis*	8	74
Understanding, acceptance	8	32
Finding a missing piece	2	4
Empowering	7	23
Counselling	7	15
*Two sides of a diagnosis*	8	32
Challenges as well as benefits	6	10
Complacency, labelling	7	22

When providing interview extracts, the following textual conventions are used:

Words omitted to shorten quote: …

Explanatory information provided by authors: [text].

### Theme 1—Barriers to a Diagnosis

As autism is a condition that must be present in childhood, it was of interest to understand more about the childhood experiences of autistic adults who are not diagnosed until later in life and understand why a diagnosis was missed in childhood. The most common reason participants believed they were not recipients of a diagnosis earlier in life was their high achievement. Almost every participant explicitly reported that their academic achievements and social successes were a barrier to receiving a diagnosis. As such, participants recounted that parents and professionals were unsure as to whether a diagnosis was indeed helpful or appropriate for a child they saw as successful in school.

My dad, he thought, what benefit would it bring? Because you ultimately weren’t developing like you were handicapped, you weren’t held back, you were actually excelling in school and whatnot. You were constantly bringing friends home and you were excelling. So what benefit would you say it is?

Many believed that when they were children there was a poor understanding of the autism spectrum, and it only included those with high functional needs. As a result, parents were concerned that having their child receive a diagnosis could discount their academic achievements and possibly put them at a disadvantage when furthering their education and professional development. Much of this was related to the fact that autistic people were understood to present in a certain way, and the participants had different presentations that made this autistic stereotype not relevant or a close match to the way they presented in everyday life. For instance, some female participants discussed being perceived as shy, which was perhaps more acceptable for females and hid their autistic characteristics. Another discussed how her female presentation of autism, which looked very different from her father's, served as a barrier to getting a diagnosis.

I remember for me personally, one of the things that took me so long to get an assessment was people would say to me, ‘How do you have such a good understanding of autism when you're on the spectrum? It doesn't make sense because of this theory of mind, because you're not supposed to have empathy and understand other people's perspectives. How do you go about it? How can you possibly understand what it's like to have autism if you're saying you're autistic?’ And I could never understand that myself.

This is a pertinent example of how strengths, such as academic ability and social skill, served as barriers for identification and treatment. It also highlights how commonly held stereotypes can directly impede identification.

For many, the lengthy process of receiving a diagnosis from a professional was a barrier in and of itself. Some expressed that multiple professionals assessed them over many months, which was emotionally draining:

For me, it was a psychological kind of torment because it was 25 years of feeling like an alien boiling down to one moment of him and his team or whoever he's working with, effectively saying, yes, I'm insane or I'm not. And I felt it was that constant pressure. And if they say no, there's actually no evidence to suggest it here, I almost fall back to square one … it was psychologically draining just because it was so much boiling down to what, one day.

Some participants opted for a private service so that they would be able to eliminate the long wait lists, and because they knew that they presented in ways that contradicted certain autistic stereotypes:

I didn't want to waste time, you know, life's too short. Yeah, I'm getting old. I didn't want to waste time or hassle with the NHS because, you know, I've done a lot of living. I speak neurotypical fluently. I don't look autistic. They’ll say ‘Well, you went to a mainstream school, you got two degrees. You're in employment, in fact, successfully self employed. Come on. Why waste time on you.’

While participants understood that services were stretched, for most, the fact that they had to not only wait for a diagnosis but repeatedly undergo testing that they feared may contradict the understanding they had come to regarding their diagnosis, was itself a barrier with significant mental health costs. In many ways, this stemmed again from the misperceptions of autistic people that extended into adulthood and the idea that an autistic adult who had a certain level of success may not equally be autistic.

### Theme 2—Negatives of a Non-diagnosis

While the participants described aspects of their childhood selves that were ‘successful’ and thus made their autistic traits less congruent with their outward-facing selves, they also described painful experiences that negatively affected their sense of self. While a few participants discussed the sensory challenges of the condition, for most, the social miscommunications were both the most indicative of autism in childhood and caused them the most issues throughout their lifespan. Many used the metaphor of feeling like an alien or foreigner, such as speaking a different language or feeling transplanted from a different world. As one participant explained, having to assimilate as an autistic person meant repressing one's native language. As she put it, ‘I speak neurotypical because I live in neurotypical land … I've lived in the neurotypical country for 60 years yet.’ Feeling alien was a reflection of the fact that other people were constantly sending participants the message that they were odd, different and hard to understand. As one explained:

The biggest memories I have is starting in primary school and literally being on the playground and feeling like I was on an alien planet. I couldn't understand these children and I didn’t understand how to interact with them, and everything they did was like a foreign language. And it was so hard. And nothing I did was ever right.

Sensitive to the adverse reactions they often received due to their socio-communicative differences, participants often camouflaged or masked their autistic traits. Camouflaging not only compounded anxiety that they were not normal, but it was exhausting. Many explicitly focused on the need to mask to be successful in what was considered the highest stake settings, professional settings or interacting with strangers.

Everyone I know, everyone acts more professional in front of important people than they traditionally would. I guess what I'm saying is, it's a whole different, whole other level … I also think, well, when that camouflage happens so often to the point where you're not thinking about it, well, that is surely part of your personality as well. And in many ways, that is now my personality.

In this way, masking led to a fragmented sense of self. As one participant explained, ‘I've always found a lot of contradictions in myself, I've never really been fully resolved. Masking is one of them, so I can be gregarious and yet not enjoy the process at the same time.’ In this sense, living without a diagnosis did not provide the justification participants needed to explain their social experiences and how their differences made them no less of a person than anyone else. Instead, a missed diagnosis heightened their need to take on neurotypical characteristics as they had no other identity they could present to the outside world. For some, masking led to severe self-harm behaviours, addiction and mental health conditions, such as anxiety and depression. It led to decreased self-esteem for all participants as they found themselves pretending to be someone they were not.

### Theme 3—Benefits of a Diagnosis

While participants struggled for years to understand why certain social situations were challenging, the ability to understand themselves as autistic for the first time as an adult after gaining a diagnosis was unanimously viewed as a life-changing, revelatory experience. Participants used several metaphors to describe how receiving a diagnosis changed their lives, illustrating how life without a diagnosis was a life without all the right pieces of the puzzle, the language, or the manual that a diagnosis now provided.

For me, it's not a label. It's a signpost. And it points to the help you need and how to look at things a different way and embrace that as a strength.

More than anything, a diagnosis was empowering as it demystified their social differences, providing a logical, scientific explanation for their experiences. Some discussed this in terms of neurology and an understanding that they are wired differently. Having the ability to learn about the condition and speak to other autistic people further enhanced their understanding of their status as ‘autistic’. They were able to explore how they were similar to other autistic people rather than simply different from most people. Some of the power that came with a diagnosis was that it allowed for compassion, both for themselves and from other people:

It was a bit like if you don't have a diagnosis or something, it kind of feels like they're out to get to you. Instead of trying to be positive, they're like, ‘Why aren't you doing this?’ And now there's less of that. And it's more like they understand that OK, I have a diagnosis, and I have a disability. And that I’m not OK with work and stuff like that. So I would say it’s less stressful.

One of the most significant forms of support that a diagnosis afforded for several participants was access to counselling. Many argued that counselling is essential for autistic people who journey to a diagnosis as adults. The benefits of counselling were two-pronged. For one, they were objective, which meant that participants were no longer relying on opinions of family or anecdotal information from people in the autistic community, which some felt marred their judgement in terms of understanding how the condition affected their life. As one participant put it, ‘A professional who comes completely objectively and tells you, ‘you have autism’ has no agenda.’ Second, speaking to a professional allowed them to be guided through their autistic journey rather than having to do this exploration entirely on their own. Almost all participants reiterated the need for more mental health supports for autistic people, specifically through counselling.

### Theme 4—Two Sides to a Diagnosis

Taking on an autistic identity was not always easy or positive, particularly as participants had arrived at the point of seeking diagnosis through the somewhat painful process of recognising how they were different from others. While the diagnosis was often helpful, getting to the point that they needed the diagnosis, such as through increased sensory issues, meltdowns, and frank conversations with parents or partners about being ‘atypical,’ was not easy. For some, the process of having to recognize things in their past that were markers of autism were challenging. One participant described being diagnosed as:

Very hard, very, very hard because you have to go in there honest with the fact that your whole history, big parts of it were a lie … And there’s these glaring issues and you see how multiple times you were failed by various professionals that should have and could have seen issues and probably felt a little bit but didn't.

While understanding the self as autistic was often helpful, it did not mean that participants were completely comfortable with their new identity. Some were afraid that they would become complacent or make excuses for themselves due to a diagnosis. For some, this meant that in retrospect, they were pleased that they hadn’t received a diagnosis earlier as they felt it would have limited their full potential. As one participant expressed, they believed that children who receive an early diagnosis may ‘now not got the strength and experience to overcome issues because previously all the issues were taken away by everybody that thinks they're helping, but really are disabling them.’

Taking on an autistic identity also meant having to navigate disclosure, which almost all participants found stressful. This largely stemmed from an understanding that autism remains for many a misunderstood, often stigmatized condition. One participant voiced his hesitation in sharing his condition with others, remarking that ‘You know, it's cruel world when you go for a job. I mean, I work for a housing charity and with the most liberal minded as it gets, but I'm still nervous about telling them.’ Remarked another participant:

‘We had a documentary on the on the television some years ago. It was about low functioning autists who aren't able to communicate and are very much disabled … There's a whole percentage of the population who now believe that all autists are like that. So when I say somewhere that my daughter has autism, I don't even tell them about me, because my daughter is more obvious because she has a speech impediment.’

In many ways, these issues were a continuation of the same stereotypes that may have resulted in participants not receiving a diagnosis as children. For instance, participants discussed how autistic people experience discrimination during hiring processes and how there are limits to how accepted autistic people are in adult professional life. Many expressed the belief that while autism research has expanded and awareness may be heightened, actual acceptance is not. Stereotypes of autistic people as unempathetic and cold, or savant or ‘low functioning,’ or simply people who bring problems to the workplace, still pervade, and this itself made participants wary of disclosure. For two participants this fuelled a desire to change these perceptions. For most, some of those stereotypes led to dissonance and a further justification for masking.

## Study 2 Discussion

The quantitative results of Study 1 indicated that as autistic adults aged they did not experience the gains in QoL as seen in neurotypicals. We investigated this further by assessing the role that age of diagnosis played in this QoL trajectory of autistic adults. In our sample, we found that those who received diagnoses later in life were experiencing more mental health challenges and reported higher levels of autistic traits. To further investigate what challenges autistic adults who received a diagnosis in adulthood faced and better understand their autism journey, we interviewed eight adults diagnosed with autism as adults who had participated in Study 1 about their experiences both living the majority of their lives without a diagnosis, and how receiving a diagnosis affected them. Their interviews uncovered four themes, all of which further enhanced our understanding of the quantitative findings.

Central to the interviews was the idea that prior to receiving a diagnosis, participants had a poor understanding of why they were different from others and that they had to hide these differences to gain acceptance and success. To keep up a façade of normalcy, avoid labels, and fulfil expectations, participants engaged in masking. Like similar research on masking in autism suggests (Hull et al., 2017), participants found the process exhausting, stressful and humiliating. Research on masking in autism suggests that it is incredibly harmful to mental health and is linked with increased depression (Cage et al., 2018) and even suicidality (Cassidy et al., 2018). Masking is also directly related to stigma. People who report more significant masking also report greater consciousness of autism stigma (Perry et al., 2021), illustrating the cyclical effect that stigma has on increasing a person’s perceived need to mask and reinforcing the idea that natural behaviors should be hidden. Indeed, participants were all well aware of autism myths, including mindblindness, rudeness, savant abilities and having intellectual impairments which they cited as impacting upon their ability to disclose, and serving as a source of stress. Understanding timing of diagnosis and masking may be an important area of future research. It could be that individuals who have been diagnosed later in life engage in more masking, which contributes to being missed for an earlier diagnosis. This may help explain why QoL outcomes are lower in this subgroup of the population, and could lead to targeted interventions for individuals diagnosed later in life.

Masking was discussed most regularly as a way to conform to neurotypical expectations and avoid negative appraisal. As discussed in detail by participants, having socially atypical behaviors in line with autistic social styles often results in being negatively appraised, such as being judged as more awkward (Grossman, 2015) and less friendly (Stagg et al., 2014) than neurotypicals. These impressions can lead to exclusion and rejection, particularly for autistic people with subtler characteristics (Griffin, 2019). To avoid this, masking is common, even though it leads to adverse mental health outcomes.

As a counter to masking, disclosing an autism diagnosis may be vital to correcting negative and inaccurate perceptions about autistic people. For instance, Matthews et al. (2015) found that college students were more accepting of hypothetical individuals with autistic characteristics when they were explicitly identified as being autistic in comparison to the same individuals with no label, and similar work by Brosnan and Mills (2016) also found these effects. As participants were undiagnosed until adulthood, it stands to reason that being judged for atypical social interaction styles without the ability to identify as autistic compounded social isolation and subsequent feelings of self-worth. The importance of a label, and the ability to counter stereotypes that they were ‘alien’ after receiving a diagnosis shows the importance of a diagnosis in and of itself. Moving forward, it may be constructive for autistic adults in possession of a new diagnosis to work with specialists on how to disclose their diagnosis so that they can be understood by neurotypicals in a way to improve double empathy (Milton, 2012).

Davidson and Henderson (2010) discussed that, similar to coming out as a sexual minority, coming out as a neurological minority carries risk for the individual who may be faced with stigma. At the same time, coming out as a minority in any sense can be liberating for several reasons. It increases contact between the neurotypical and autistic world, which improves autism understanding and reduces stigma (Corrigan & Matthews, 2003). That is not to say that disclosure is something that should be taken lightly or something forced on an individual. As Davidson and Henderson (2010) highlighted, a great deal more research on how to disclose should be conducted, including the need to highlight strengths and provide explanatory information. In a recent review by Thompson-Hodgetts et al. (2020), it was found that disclosure carries risks that may lead to many autistic people to not disclose, including autism becoming a primary identity, and resulting in stigma and discrimination. Indeed, the review found that disclosure is often viewed as beneficial by researchers and professionals but is not considered quite so favourably by autistic people themselves. It shows the importance of giving a voice to autistic experiences to understand this complex process. Future work may want to investigate experiences of disclosure and timing of diagnosis and the effects of disclosure on masking. It may also want to examine the impact of autism-community based counselling to help newly-diagnosed adults navigate these complex issues (Crane et al., 2021).

## General Discussion

In summary, we found in Study 1 that autistic adults, unlike neurotypicals, were not experiencing gains in QoL as they aged. Upon closer examination, we found that this was related to age of diagnosis, with those diagnosed as autistic later in life experiencing poorer QoL and higher autistic traits. We also found that gender played a role in this, with women receiving a later diagnosis and reporting higher autistic traits. In Study 2, we investigated how QoL and late diagnoses are experienced by adults diagnosed with autism in adulthood. While receiving a diagnosis was often a positive experience, the diagnostic process and the years lived without a diagnosis were not always easy. In particular, the interviews revealed a need to improve the process of receiving a diagnosis as an adult, including the availability of therapy during and after diagnostic testing, and more broadly, how masking and autistic stereotypes can damage a person’s sense of self and impact disclosure.

It is important to note that our sample consisted of individuals who self-reported an autism diagnosis without formal testing that confirmed this to be the case. While research suggests that reporting the age of diagnosis and that it was by a medical professional are reliable indicators of a true diagnosis in a study on parents of children with autism (Daniels et al., 2012), this method would benefit from further validation in an adult sample.

Future research may also want to employ purposive sampling and directly compare QoL and AQ scores in adults who received a diagnosis at specific time points in the lifespan (i.e. in early childhood, late adolescence, middle adulthood etc.). In keeping with a lifespan developmental theoretical framework, identity is influenced differently at different time points of development (Topolewska-Siedzik & Cieciuch, 2019). It would be interesting to understand how people of different ages conceptualize their autistic identities at these other time points and how receiving a diagnosis versus pre-possessing a diagnosis at these ages influences outcomes. Indeed, it may be that there are aspects of receiving a diagnosis that are universal regardless of age. For instance, Mogensen and Mason (2015) interviewed autistic teenagers on how a diagnosis affected their sense of self, and the themes are strikingly similar to the themes found in Qualitative Study. This suggests that autistic individuals may benefit from services that allow them to explore issues relating to possessing a neurodiverse identity irrespective of age. It may also be helpful to understand how an understanding of autistic identity changes over time following a diagnosis.

Similarly to Mogensen and Mason (2015), our participants in Study 2 were recently diagnosed, and in a sense, still coming to terms with their autistic identity. Future studies would be of interest to understand how individuals assimilate an autistic identity as they go through life. This may be particularly important regarding tailoring support services for individuals at different time points following a diagnosis.

Another limitation is that our results in many ways represent a ‘snapshot in time’ as services for providing early identification and support for autistic children continuously improve. However, there remain inequalities in identification regarding gender, ethnicity and socioeconomic status (Mazurek et al., 2019). Future research will want to continue researching this topic with younger birth cohorts, who presumably will be diagnosed with autism at an earlier age and with an improved understanding of autistic symptomology. It may be that the future generation of autistic and TD adults will show similar age effects on QoL and AQ profiles considering this increased understanding of autism, earlier identification and better access to supports.

This research has revealed a number of issues with regards to late diagnosis in autistic adults, many of which require further study and recommendations to improve current services. First, our results from Study 1 do not suggest that those with a late diagnosis are in any less of a need of diagnosis than those diagnosed earlier. Indeed, adults diagnosed later in life reported higher AQ scores and lower QoL, indicating that they have not evaded diagnosis due to a less severe set of autistic symptoms or better adjustment. This trend is very much reflected in the qualitative data. Participants described childhoods where they experienced social difficulties in line with what we know affects autistic children diagnosed at an earlier age; they experienced increased rates of social exclusion, bullying, and mismatches in communication. Quantitative results suggest a better quality of life the earlier a person was diagnosed, and qualitative results suggest that despite academic and professional success, participants were painfully aware of how they struggled socially without knowing why, and this led in many instances to depression anxiety and even victimization.

Future work will want to investigate further what types of individuals are most at risk for a missed diagnosis. Our quantitative results suggest women were far more likely to be diagnosed as adults than males, in keeping with research that indicates that females with autism are often missed for an earlier diagnosis due to camouflaging (Dean et al., 2017; Lai et al., 2016). That said, while camouflaging may contribute, it may not be the entire reason autistic females are often ‘missed’ with regards to an early diagnosis. Given past research such as the ‘male brain theory of autism’ (Baron-Cohen, 2002), there may be institutional barriers that affect women’s ability to be perceived as autistic by professionals, educators and even parents. Further work investigating how gender affects identification and how to improve services for women of all ages who may be on the spectrum is imperative. In the same way that women were not identified early in life at the same rate as males, our qualitative results suggested that children presented as being successful were also at risk for being undiagnosed. Understanding the misconceptions regarding achievement and autism will be an important initiative, particularly in light of our findings that missed diagnoses contribute to mental health issues.

As the interviews reveal, receiving a diagnosis is highly emotional. It involves a reconceptualization of who you are, how you explain yourself to other people, and how you rewrite your own story. Almost every participant talked about the importance of therapy and the difficulty in receiving treatment, with limited sessions and long waits for services. There needs to be perhaps a realization that adults receiving late diagnoses of autism will be dealing with the residuals of years of trauma, of years of being misunderstood, criticized and bullied without knowing why. As suggested by our interviews, receiving a diagnosis allows everything to finally ‘make sense.’ Still, this involved reflecting on a life without a diagnosis, and redefining who you are, tasks that require a great deal of emotional fortitude. Throughout this process, accessing mental health services is vital, as is more research investigating how to improve the QoL in adults who receive late diagnoses. Our qualitative data suggested several routes. One is the need for individual counselling, which many cited as helpful but limited. Another is the ability for autistic adults with late diagnoses to meet other autistic adults. Several participants discussed the ease of talking to other autistic people, who they believed understood them on a deeper level than they experience with many NTs. Thinking about how counselling services and support groups for newly diagnosed adults could be improved is an important area of future research.

Another area of continued research should be the adult autism diagnostic process's effect on mental health. Participants described stressful, arduous diagnostic procedures for adults. Many discussed choosing to pursue a private diagnosis, as a publicly funded avenue involved years of waiting. All participants discussed the required lengthy repeated testing and having to liaise with parents and partners about aspects of yourself that were hard to talk about, which often involved reflecting on personal struggles and reliving painful memories. Understanding more about the adult diagnostic process and how to make diagnoses and support services affordable, efficient and humane is essential. Current research suggests that diagnostic procedures for adults are poor across countries compared to those of children, which includes a lack of formal support services (Huang et al., 2020). A lengthy diagnostic process with little support along the way may be, in and of itself, a contributing factor to the poorer QoL for adults diagnosed later in life and should be investigated. More research should focus on how we can create a more expedient diagnostic procedure for adults, including self-assessment in the early stages of diagnosis, and assistance for mental health issues while waiting for a diagnosis.

Finally, both studies suggest that there are aspects of receiving a diagnosis early in life that is protective regarding mental health. One possibility suggested by Study 2 with regards to the benefits of a diagnosis is that those who are diagnosed later in life are primarily using their diagnoses as a tool to understand themselves, not to seek supports that would improve their QoL, specifically with respect to their autistic traits. For instance, children diagnosed with autism early in life qualify for specific accommodations and are eligible for services, in line with a shift towards educational inclusion (Farrell & Ainscow, 2002). This means that children diagnosed at an early age are receiving targeted supports early on that increasingly improve outcomes, which may in part explain higher QoL in those with early diagnoses (Flanagan et al., 2012; Granpeesheh et al., 2009; Itzchak & Zachor, 2011).

Creating similar opportunities for adults, including funding therapeutic services where individuals can be guided on disclosure, identity, masking, anxiety, and sensory issues, is critical. Compared to the services available to children with autism, autistic adults have very little available funding support and assistance (Camm-Crosbie et al., 2019). While the years lived without a diagnosis may continue to impact QoL, research such as this highlights that this is a portion of the autism population in current need of supports, though they are in many ways the least supported. Some preliminary research on autistic-led therapy for newly diagnosed adults suggests that this could be a helpful avenue to develop further, as participants reported increased understanding and knowledge when interacting with autistic peers (Crane et al., 2021). More research is needed on the efficacy and feasibility of these types of support groups and how to tailor general counselling practices to address the needs of newly diagnosed autistic adults.

As this research suggests, autistic adults receiving a diagnosis later in life are particularly vulnerable due to the cost of living for so long without a diagnosis. Creating supports for this specific portion of the autistic population is vital if they are to experience the same gains in QoL as they age in relation to their NT peers. As revealed in the interviews, there is hope in that receiving a diagnosis is empowering. It gives autistic adults a chance to reclaim portions of their history in which they were misunderstood. More work understanding how to use a strengths-based approach and an identity-formation framework in working with newly diagnosed autistic adults would be critical for future research.

## Supplementary Information

Below is the link to the electronic supplementary material.

Supplementary file1 (DOCX 17 kb)

Supplementary file2 (DOCX 24 kb)
