# Supplementary material for: Autism Through the Ages: A Mixed Methods Approach to Understanding How Age and Age of Diagnosis Affect Quality of Life
Source: J Autism Dev Disord. 2021 Sep 4;52(8):3639–54. doi: 10.1007/s10803-021-05235-x (PMC9296439; doi:10.1007/s10803-021-05235-x)
Supplement: Supplementary file 1 — Supplementary file1 (DOCX 17 kb) [file 10803_2021_5235_MOESM1_ESM.docx]

**Supplementary Materials**

Table A shows descriptive statistics of the variable of interest divided between ASC and TD and by sex. All variables were not normally distributed as indicated by the Kolmogorov Smirnov tests reported in Table A. Table B shows the correlations between the five quality of life measures.

**Table A.**

*Median (and Range) of Age, Age of Diagnosis, Loneliness, Social Anxiety, Social Avoidance, Social Support and Life Satisfaction Divided Between ASC and TD Group and by Sex*

|  | *Kolmogorov Smirnov test* | *ASC* | | *TD* | |
| --- | --- | --- | --- | --- | --- |
|  |  | *Male* | *Female* | *Male* | *Female* |
| *N* |  | 101 | 107 | 78 | 113 |
| *Age* | KS(399) = .13,  *p* < .001 | 24 (18-57) | 27 (15-63) | 26.5 (16-73) | 33 (18-79) |
| *Age of Diagnosis* | KS(208) = .13,  *p* < .001 | 15 (2-54) | 21 (3-62) |  |  |
| *Loneliness* | KS(399) = .05,  *p* = .02 | 52  (20-79) | 58  (22-79) | 42.5  (20-79) | 44  (21-75) |
| *Anxiety* | KS(399) = .06,  *p* = .001 | 44  (0-68) | 49  (10-71) | 29  (0-55) | 29  (1-66) |
| *Avoidance* | KS(399) = .07,  *p* < .001 | 38  (0-67) | 43  (2-72) | 23  (0-46) | 19  (0-72) |
| *Support* | KS(399) = .07,  *p* < .001 | 56  (12-84) | 58  (12-84) | 65  (14-84) | 69  (26-84) |
| *Satisfaction* | KS(399) = .07,  *p* < .001 | 18  (5-33) | 17  (5-35) | 23  (10-35) | 23  (6-35) |

**Table B.**

*Spearman’s Correlations Coefficients of Relationship Between Loneliness, Social Anxiety, Social Avoidance, Social Support and Life Satisfaction.*

|  | *Loneliness* | *Anxiety* | *Avoidance* | *Support* |
| --- | --- | --- | --- | --- |
| *Loneliness* |  |  |  |  |
| *Anxiety* | .496* |  |  |  |
| *Avoidance* | .485* | .862* |  |  |
| *Support* | -.625* | -.310* | -.375* |  |
| *Satisfaction* | -.561* | -.362* | -.393* | .543* |

* *p* <.001

We explored the effect of diagnosis and sex on the five quality of life measures separately. Although the data are not normally distributed, two-way ANOVAs with diagnosis (ASC and TD) and sex (male and female) as between-subjects factor was conducted due to the absence of a non-parametric equivalent. The F-test has been found to be robust to violations of normality in terms of Type I error (Blanca et al., 2017; Schmider et al., 2010).

There was a significant main effect of diagnosis on Loneliness (*F*(1, 395) = 74.17, *p* < .001, *η_p_^2^* = .16). Post-hoc tests (Bonferroni corrected) showed that Loneliness scores were significantly higher in the ASC group than in the TD group (*p* < .001, 95% CI 8.85, 14.08). There was no significant main effect of sex on Loneliness (*F*(1, 395) = 2.83, *p* = .09, *η_p_^2^* < .01). There was also no significant interaction effect between diagnosis and sex on Loneliness (*F*(1, 395) = .01, *p* = .91, *η_p_^2^* < .01).

There was a significant main effect of diagnosis on Anxiety (*F*(1, 395) = 121.42, *p* < .001, *η_p_^2^* = .24). Post-hoc tests (Bonferroni corrected) showed that Anxiety scores were significantly higher in the ASC group than in the TD group (*p* < .001, 95% CI 12.71, 18.23). There was also a significant main effect of sex on Anxiety (*F*(1, 395) = 7.52, *p* = .006, *η_p_^2^* = .02). Post-hoc tests (Bonferroni corrected) showed that females had higher Anxiety scores than males (*p* = .006, 95% CI 1.09, 6.61). There was no significant interaction effect between diagnosis and sex on Anxiety (*F*(1, 395) = .25, *p* = .62, *η_p_^2^* < .01).

There was a significant main effect of diagnosis on Avoidance (*F*(1, 395) = 135.75, *p* < .001, *η_p_^2^* = .26). Post-hoc tests (Bonferroni corrected) showed that Avoidance scores were significantly higher in the ASC group than in the TD group (*p* < .001, 95% CI 13.91, 19.56). There was no significant main effect of sex on Avoidance (*F*(1, 395) = 3.33, *p* = .07, *η_p_^2^* < .01). There was also no significant interaction effect between diagnosis and sex on Avoidance (*F*(1, 395) = 2.83, *p* = .09, *η_p_^2^* < .01).

There was a significant main effect of diagnosis on Support (*F*(1, 395) = 32.88, *p* < .001, *η_p_^2^* = .08). Post-hoc tests (Bonferroni corrected) showed that Support scores were significantly lower in the ASC group than in the TD group (*p* < .001., 95% CI 6.14, 12.54). There was no significant main effect of sex on Support (*F*(1, 395) = 2.76, *p* = .10, *η_p_^2^* < .01). There was also no significant interaction effect between diagnosis and sex on Support (*F*(1, 395) = 2.90, *p* = .09, *η_p_^2^* < .01).

There was a significant main effect of diagnosis on Life Satisfaction (*F*(1, 395) = 47.86, *p* < .001, *η_p_^2^* = .11). Post-hoc tests (Bonferroni corrected) showed that Life Satisfaction scores were significantly lower in the ASC group than in the TD group (*p* < .001, 95% CI 3.64, 6.52). There was no significant main effect of sex on Life Satisfaction (*F*(1, 395) = .01, *p* = .99, *η_p_^2^* < .01). There was also no significant interaction effect between diagnosis and sex on Life Satisfaction (*F*(1, 395) = .10, *p* = .76, *η_p_^2^* < .01).

In summary, there was a main effect of diagnosis on all five quality of life measures, with the ASC group having higher Loneliness, Social Anxiety and Social Avoidance scores, and lower Life Satisfaction and Social Support scores compared to the TD group. In contrast, there was a main effect of sex on Social Anxiety only, with females having higher scores than males. Finally, there was no interaction effect between diagnosis and sex on any of the five quality of life measures.

**Table C.**

*Spearman’s Correlations Coefficients of the Relationship Between Age and the Five Quality of Life Measures (Loneliness, Social Anxiety, Social Avoidance, Social Support and Life Satisfaction) for the TD group, and Spearman’s Correlations Coefficients of the Relationship Between Age, Age of Diagnosis and the Five Quality of Life Measures for the ASC group.*

| *Group* | *Variable* | *Loneliness* | *Anxiety* | *Avoidance* | *Support* | *Satisfaction* |
| --- | --- | --- | --- | --- | --- | --- |
| *TD* | Age | -.321*** | -.250*** | -.225** | .345*** | .120 |
| *ASC* | Age | -.050 | .078 | .082 | -.026 | .034 |
| *ASC* | Age of Diagnosis | .131 | .203** | .235** | -.139* | -.095 |

* *p* < .05, ** *p* < .01, *** *p* <.001

Two-tailed Spearman’s correlations were conducted to explore whether participants’ age was related to the five quality of life measures. Correlations were performed separately for those with diagnosis of ASC and TDs to test whether older participants in both the ASC and TD group have higher quality of life. As Table C shows, age positively correlated to social support and life satisfaction, and negatively correlated to loneliness, social anxiety and avoidance in the TD group. In contrast, age did not correlate with any of the five quality of life measures in the ASC group. This suggests that several aspects of quality of life of TD individuals improve as they age, meanwhile this do not occur for people with a diagnosis of autism. Furthermore, age of diagnosis positively correlated with social anxiety and avoidance, and negatively correlated with social support. This suggests that those individuals who received their diagnosed later in life had poorer quality of life in the aspect of their social anxiety, avoidance and support.

**Table D.**

*Presents an indicative selection of quotes representing master and sub themes.

**NOTE TO PRODUCTION – as this table contains quotes which are fairly long it is best presented in landscape, therefore we have included this as a seprate file, in production please place it here ***

**Interview Protocol**

**Diagnostic process**

Remind me again what age you were diagnosed.

What made you seek a diagnosis?

Can you tell me a bit about your process in getting a diagnosis?

What has changed now that you have a diagnosis?

*Has anything changed for the better?

*Has anything changed for the worse?

How were things different before you had a diagnosis? (positives and negatives)

**Childhood**

Can you tell me about what your childhood was like without a diagnosis?

What were the supports like then for people with autism? What about awareness?

Why do you think you weren’t diagnosed earlier in life?

*What’s different about your childhood and your child’s, given that they had an earlier diagnosis? (if they have mentioned autistic child)

**Gender**

How do you think your gender played a role in recognizing you had autism?

Did it impact your diagnostic process? If so, how?

**Mental health**

Can you tell me about how you feel receiving a diagnosis has impacted your mental health?

Can you tell me about any positive or negative impacts of receiving a diagnosis of your mental health

**Social supports**

Who are the people in your life who you feel support you the most?

What does that support look like?

Has having a diagnosis changed the way your social supports understand you?

What do you think would make the biggest difference in your life with regards to getting support for autism? (ie services, financial supports, societal changes)

**Identity**

How do you think getting a diagnosis has changed the way you think about yourself?

How do you think it might have changed the way other people think about you?

Are you familiar with the term masking or camouflaging? *it refers to hiding one’s symptoms to fit in with neurotypicals

Is this something that you ever find yourself doing? If so, can you tell me a bit about some of the times when you have masked your autism? And what that felt like?

**Conclusion**

What do you think is most misunderstood about autistic people?

What do you think are the greatest strengths you have in relation to your autism?

What are some of the ways that autism makes you proud of who you are?
