# Supplementary material for: Autism Through the Ages: A Mixed Methods Approach to Understanding How Age and Age of Diagnosis Affect Quality of Life
Source: J Autism Dev Disord. 2021 Sep 4;52(8):3639–54. doi: 10.1007/s10803-021-05235-x (PMC9296439; doi:10.1007/s10803-021-05235-x)
Supplement: Supplementary file 2 — Supplementary file2 (DOCX 24 kb) [file 10803_2021_5235_MOESM2_ESM.docx]

| Themes/Subthemes | Examples | | |
| --- | --- | --- | --- |
| Barriers to a diagnosis |  | | |
| Academic and social success |  | | |
|  | ‘I believe that my mother didn't want to press the issue of me having any disorder because she feared that I might need a special school and have no chance of any professional life. Because from the special school, you couldn't get a tutor or A-levels. Yeah, you couldn't go to university. And she very much wanted us to go to university. So I think that was the main motivation for her. She didn't want to jeopardise my academic future at the time.’ | | |
|  | ‘In primary school, I was the best in the class by quite a long way and also was a prefect as well. So, I mean, I was a bit odd, and I sometimes I wonder if I am actually Asperger's because I've never really had any problems making friends. And I certainly never any problems making friends at that age… I just didn't really stand out in a negative way. And, of course, you know, the implication being that the diagnosis of anything is a bad thing.’ | | |
| Perceptions and Stigma |  | | |
| Gender (for females) | ‘My whole family is Greek. And the culture there is a bit different. It's just lack of understanding and stuff like depression, anxiety or whatever… And I think to have something like a diagnosis is technically in their eyes like there's something wrong with me because they're so scared. A little like they just didn't want me to be stamped or branded.’  ‘It was a conscious choice [to not be diagnosed]. It probably helped that my mum was a qualified social worker and she also has a master's in psychology. I think in her mind she knew, and that was all that was really needed. I think her opinion was that it wasn't necessary to put that label on me until I was old enough to decide for myself.’ | | |
| Long, difficult process | ‘If I was a boy, I think I would have been forcefully diagnosed at 13. It’s well documented, isn’t it, that girls are often missed because they generally are quiet or they come across as shy. They have a lot of qualities that match and so I suspect that's why it was missed in childhood when school would have intervened. And it must have been overlooked in secondary too because I'm starting to look back and wonder how nobody noticed. My own assumption is that they just put it down to being a girl. That's all I can assume.’ | | |
| Negatives of a non diagnosis | ‘They told me to wait until I was five years plus. And by that point, because it was such a revelation, it was causing torment on a daily basis, knowing I was going to have to wait. Five years of struggling.’  ‘And all through the process, I had to see three or four different people like going up a hierarchy of consultants. See one, and then they say no, yes. Then see the next one, the next one, the next one, and then the fourth one who’s the boss who, again, diagnosed me within about a minute. So, you know, by that time, it was fairly self evident to me that I had many of the traits of autism.’ | | |
| Feeling like an alien |  | | |
| Not fitting in | ‘I could think back to so many times when I felt like an alien on another planet. I just couldn’t understand what was going on.’  ‘For years, I’d felt like somewhat of an alien for years and never really made that connection in that sense because I didn't understand autism very well.’ | | |
|  | ‘Everything I did was just always so off the mark, and it’s funny because I think a lot of understanding of autism is that we don't have empathy. But for me, I did have empathy, but it killed me. So I would always realise that I’d so missed the mark. I could see every time someone rolled their eyes, or I was scared that I could see that, but couldn't understand why. I couldn't see what I'd done wrong and how I'd missed, and I was so trying to get it right.’ | | |
| Bullying/Exclusion |  | | |
|  | ‘Mockery was always a thing because I was like something of a circus freak and memorised textbooks… People were always mocking me in other situations. I was different. I definitely didn't have any, like, close friends. I wasn’t cool to talk with during breaks. I was there when they needed something.’  ‘Well, in jobs and in that sort of setting, yeah, where I had no idea what people were saying and they had no idea what I was saying, and it could be an absolute nightmare. And I was bullied remorselessly, but never by children. Never at school. Yeah. But by people in authority and that sort of thing. So it's not a not nice, not nice thing to have to deal with, especially in a place of work where you're trying to do a job.’ | | |
| Masking/camouflaging |  | | |
| Mental health strains | ‘In the completely new environment with new people, I'm definitely trying to act like a human being, but I usually don't hold it very long because it's exhausting. It's very exhausting. Yeah, kind of constantly watching myself. I usually blurt out something really stupid, like when I'm so concentrating on being the normal human being that I do complete overkill.’  ‘I am very secretive or private about sharing that I have autism, and I only do with people I'm comfortable with. And because of that, I kind of have a fake personality, but more like a guard or wall. I don't really show truly how I am. I don't to because then the more I show myself, the more of the autism comes up.’ | | |
| Benefits of a diagnosis | ‘My mental health has been horrendous all my life. So, you know, it's always been there ever since I was five years old. Yeah. So I suspect, you know, I initially put it down to sort of congenital depression on my mom's side. And that may be the case. But I also have watched a lot of these huge triggers. And accept it's probably is a link between being a kind of depressed kind of character, which I am depressed, I mean, I've always been cynical, depressed character all my life, and maybe being autistic, I don't know.’ | | |
| Understanding, acceptance |  | | |
| Finding a missing piece | ‘It doesn't have to change anything, but the more knowledge about it, more knowledge, in general, is always a good thing. I don't feel like I'm thinking, ‘Oh, I can't do that because I've been diagnosed,’ because that's nonsense. I'm never going to be a massive fan of public speaking, but whether I was diagnosed or not, that was always going to be the case anyway. It's just that now I know why.’  ‘When I was like fifteen, fourteen, twelve I used to think that I was planted here from the aliens. I like sci fi, I grew up with Star Trek, and there was this Mr. Spock. And he was unemotional and really logical. He always said what he meant, what was on his mind. And I was like, ‘Oh my God, he's cool. And he's an alien. And it's because of that, that he’s so different from the humans.’ And they reacted to him sometimes the same way that people would react to me. Like the Doctor McCoy, he would always rage at something that Spock said, that it was inhuman. And Spock would always remain cold and said, ‘Of course, it's inhuman, I'm not human, I’m Vulcan.’ And so I was like ‘Ah if only I was a Vulcan.’ So this was like this fantasy that I was planted here by the aliens. But when I realised that I was actually certifiably different, it was ‘OK, not Vulcan. Asperger’s. OK, I take that.’ | | |
| Empowering | ‘No question, the jigsaw finally fit. It's an analogy I been told. And I can see where that comes from. Yeah, it was always like there was a completely missing piece that I could never really account for. And after having that confirmation and learning a lot more about it, it finally made sense.’  ‘I mean, relief is much too weak a word. It was a revelation, a revelation, really. I mean, it was really everything falling into place.’ | | |
| Counselling | ‘It's helped me to understand myself, whereas before I was like, ‘I just don't understand what people are saying and doing and how to fit in.’ And now I get it, and it's OK. And it’s also have given me the ability to be able to say ‘I don’t have to fit in. I can just be me.’’  ‘In many respects, it least helps me to say ‘Oh, no, hang on, well, you are different. You are going to feel this way.’ Or if I get really wound up about having to go and see someone, because I get really anxious about it to the point of getting quite aggressive about it, at least I understand why I’m doing it, so I’d say its helped me actually. Because for me, understanding the logic behind something happening is everything, because that's the way I think. It provides an immediate, logical explanation. And there's plenty of information I can get out there now to find out how it manifests itself’ | | |
| Two sides of a diagnosis | ‘Most of the time anyways, there are some people who just want to be heard or have someone to talk to or, for example, like you and me talking. Now, I think for me that's kind of like stimulating in a way, because you're asking me stuff and I get to tell you, instead of me having to control the conversation. You're basically controlling the conversation for me by asking me stuff, and then I can just reply, you know what I want.’  ‘There needs to be some mental health support for it, like one to one and then maybe building into small classes…I have been to the GP to ask for these things, and I was told it may be more than a year or two before we can even get you on a counselling waitlist or something, and I just thought, that's not worth it, I’ll just leave it. It’s not that bad.… You have to be realistic: if someone’s suicidal, they're going to come first. But how many people with something like autism will end up suicidal because they weren't able to get the help earlier? So [we need] funding for individual therapy and mental health.’ | | |
| Challenges as well as benefits |  | | |
| Complacency, labelling | ‘[When my husband agreed with me that I may be autistic] I had a huge, bad reaction to it. Even though I always knew I was autistic, I think because somebody else knew you were, it's like I had nothing to hide behind anymore. I had to accept it once it was spoken out loud… Because all of a sudden it was out there and I couldn't hide behind them anymore. And it was a really intense feeling for two or three days. And then it was OK. And I could sort of accept it, but there was still this sort of grieving process for accepting that I had autism.’  ‘Yeah, huge relief, but also anxiety, because now it's a case of what does that mean? What does that bring with it? Because the last thing that I ever want to do is start excusing behaviours, because you don't know it's just because of autism, because that's just not an appropriate reaction. So it was also wondering, well, what comes next?’  ‘Complacency is a big one, I feel as though it would be easy to use autism as an excuse for behaviours, and how this could drive people away. Equally simply accepting something as autism might put you in the mindset of not being able to control or change it so you accept it rather improve it.’  ‘Labelling has been a big concern, and a couple of times I have also noticed certain clients, when they found out, talk and act differently around me. Most do not know as I don’t want to tarnish any work with them.’ | | |
|  | |  |  |
